# Modified sol-gel process for synthesis of molybdenum oxide-doped titanium dioxide

**DOI:** 10.1016/j.mex.2022.101742

**Published:** 2022-05-27

**Authors:** Mousab Salaheldeen Mirghani, Abdelbagi Osman

**Affiliations:** Department of Chemical Engineering, Collage of Engineering, Najran University, Najran, Saudi Arabia

**Keywords:** Modified sol-gel method, Titanium dioxide, Photocatalysis, Molybdenum doping

## Abstract

A modified sol-gel method was developed for the synthesis of pure and transition-metal-doped titanium dioxide. The process involves the hydrolysis of titanium tetraisopropoxide, which was used as a catalyst precursor, and molybdenum chloride, which was used as a doping agent. The shape and size of the final product were characterized by scanning electron microscopy, and the catalyst activity for the photocatalytic degradation of methylene blue in aqueous solutions was tested. The results indicate significant improvements in both the morphology and performance of the catalyst synthesized by the proposed method when compared to those obtained via the conventional approach using the same materials and quantities.

• The main highlights of the proposed method are as follows.
• Utilization of a double-jacketed cooling system to control and prevent temperature fluctuations during hydrolysis.
• Ultrasonication during the reaction minimizes particle agglomeration during nanoparticle formation.
• The use of two different alcohols and separation into two mixtures were experimentally demonstrated to delay gel formation, and hence, morphologically homogeneous catalyst nanoparticles were achieved.

## Graphical abstract

**Figure:**
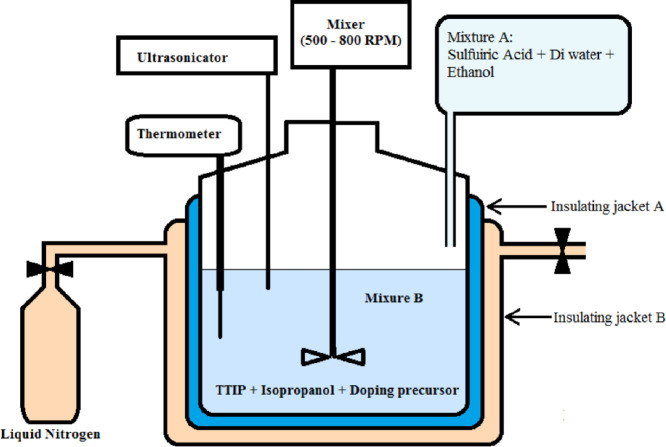

Specifications table

**Table:** 

Subject Area:	Chemical Engineering
More specific subject area:	Reaction Engineering
Method name:	Modified sol-gel process for synthesis of metal-doped titanium dioxide
Name and reference of original method:	M. S. Mirghani, “Vanadium doped titania nanoparticles for photocatalytic removal of heavy metals from aqueous solutions,” *J. Exp. Nanosci.*, vol. 16, no. 1, pp. 51–61, Jan. 2021, doi:10.1080/17458080.2021.1886277.
Resource availability:	*Not Applicable*

*Method details

The synthesis of titanium dioxide has been widely investigated by different methods, including solid-phase synthesis, such as sputtering, gas-phase synthesis, such as chemical vapor deposition (CVD), and liquid-phase synthesis, such as the sol-gel method [1], [2], [3], [4].

Among these TiO_2_ production processes, the sol-gel method has been proven to be highly effective owing to its simplicity and low-cost setup. However, the ability to control the properties of the final material is limited because of many factors, such as rapid gel formation, temperature fluctuations, and aggregate formation [5,6].

To overcome the limitations of the conventional sol-gel process, a modified sol-gel method was developed to provide an enhanced strategy for the synthesis of pure and metal-doped titanium nanoparticles. These modifications include:(1) the use of a double-jacketed cooling system to prevent rapid gel formation by precisely controlling the reaction temperature, (2) the utilization of ultrasonication during the hydrolysis period to prevent particle agglomeration, and(3) the use of two different alcohols in mixtures A and B (detailed in the section below), as it was experimentally determined to be effective in producing smaller and spherical catalyst particles [6,7].

## Stage 1: preparation of reaction setup

The reaction vessel was prepared using a batch reactor and a high-torque stirrer enveloped by a closed water-cooling system (jacket A). Another cooling system of circulating liquid nitrogen was added (jacket B), which includes inlet and outlet valves to allow the precise control of the reaction temperature within the reaction vessel. The reaction temperature was maintained in the range of 4–6 °C and monitored using a thermometer. An ultrasonication probe was inserted into the vessel from a hole in the top cover of the vessel. The role of ultrasonication is to maintain high dispersion in the mixture to prevent potential particle agglomeration.

## Stage 2: preparation of reaction mixtures

Two mixtures were prepared: mixture A consisted of sulfuric acid (0.1M H_2_SO_4_), deionized (DI) water, and ethanol in a ratio of 1:5:12.5, respectively. Mixture B contained titanium tetraisopropoxide (TTIP), the doping precursor (2500 ppm molybdenum chloride), and isopropanol at a ratio of 1:1:5, respectively.

Mixture B was placed in the reaction vessel, and the stirrer was adjusted at 500 rpm; mixture A was then added dropwise (at an approximate rate of 1 mL/s) to mixture B, and the stirring speed was raised to 800 rpm to avoid the potential stirring resistance during gel formation. All materials and the corresponding amounts used in the method are listed in Table 1.

**Table 1:** List of materials and quantities used.

Material	Quantity (mL)	Provider	Purity
Sulfuric acid	4	Sigma-Aldrich	10 % (V/V)
DI water	20	Loba Chemicals	99.995 %
Ethanol	50	Loba Chemicals	99.5 %
TTIP	5	Sigma-Aldrich	99.99%
Molybdenum chloride	5	Sigma-Aldrich	99.9 %
Isopropanol	25	Loba Chemicals	99.5 %

*Note:* When doping different transition metals onto TiO_2_, it is necessary to calculate the appropriate concentration and amount of the precursor based on the targeted doping percentage. In the case of the synthesis of pure TiO_2_, the amount of the doping metal solution was replaced with DI water to maintain the ratio of hydrolysis agents to the catalyst precursor.

## Stage 3: post-synthesis treatment

After the reaction was completed (that is, a clear gel was formed), all jackets and instruments (stirrer, ultrasonicator, and thermometer) were removed from the main reaction vessel, and the gel was decanted into a 250 mL Pyrex beaker and placed in an oven at 75  °C for 2 h. Subsequently, the oven temperature was raised at a rate of 10  °C/h for 3 h until complete drying. The resulting solid material was crushed into a fine powder and placed in a ceramic bowl for calcination.

Subsequently, the calcination process was performed in a tubular furnace in an inert environment (Ar gas was continuously passed through the furnace during the calcination process). The furnace temperature was set at 250 °C, and the ceramic bowl containing the catalyst was placed in the middle of the tube for 30 min. The temperature of the furnace was then raised at a rate of 50 °C per 30 min until the calcination temperature reached 450 °C. The furnace was then switched off; the sample was left inside the tube to cool to 25 °C, and the sample was then characterized.

Note: Temperature-controlled heating was used in the drying stage to avoid any loss in the product yield, as the reaction is in the liquid phase at this stage of the process and the gel formation does not indicate a complete reaction. An inert gas environment and temperature-controlled heating were used in the calcination stage to minimize the potential coke formation from hydrocarbon residues from the reaction.

## Method validation

To validate the effectiveness of this method compared with the conventional sol-gel process for the production of metal-doped TiO_2_, two samples of MoO_3_-doped TiO_2_ were prepared according to each method. All materials and the corresponding amounts used were the same for each method, as shown in Table 1.

**Fig. 1:**
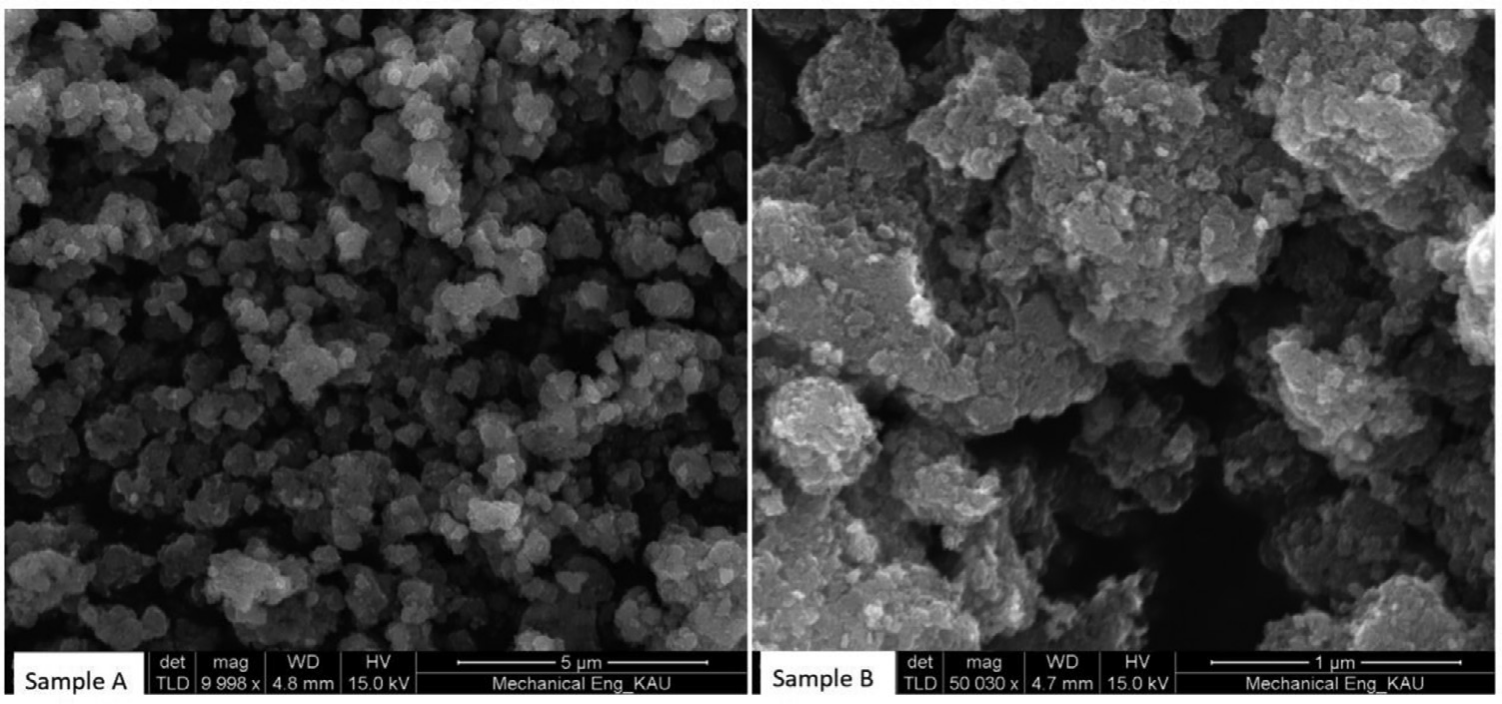
SEM images of: A)MoO_3_-doped TiO_2_ prepared by the modified Sol-gel method, and B) MoO_3_-doped TiO_2_ prepared by the conventional Sol-gel method.

**Fig. 2:**
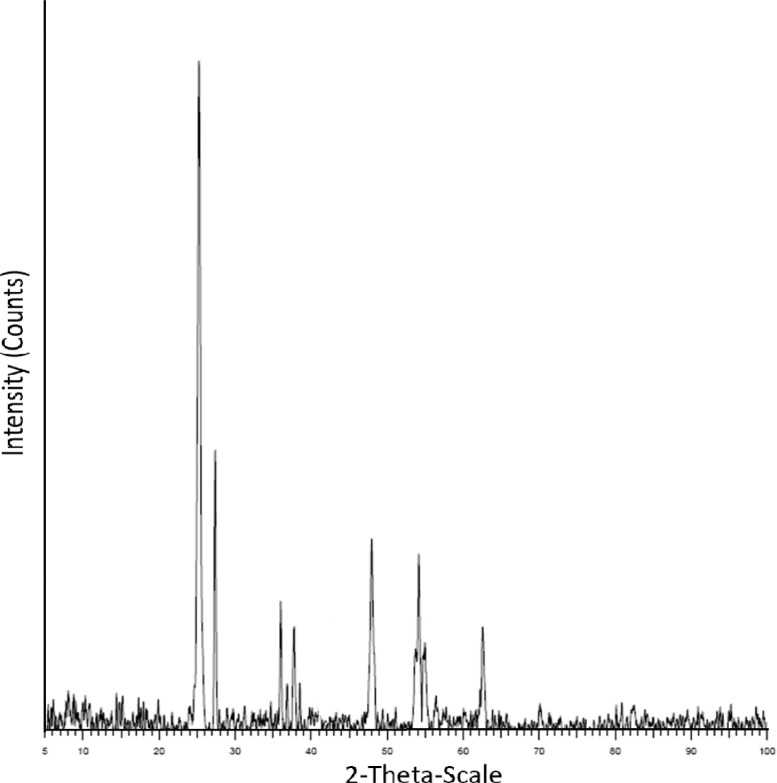
XRD pattern of MoO_3_-doped TiO2

Scanning electron microscopy (SEM Model: JOEL JSM-6460 LU) analysis was performed to investigate the morphology (in terms of shape and size) of the two samples, and the results are shown in Fig. 1. Furthermore, X-Ray diffraction (XRD Model: BRUKER TXS) analysis was carried out to investigate the phase of the catalyst and the presence of metals oxides, and the results are shown in Fig. 2.

The results for the sample prepared via the modified sol-gel method show significant improvements in terms of the shape and homogeneity of the catalyst particles (Fig. 1-Sample A), compared to the catalyst obtained via the conventional sol-gel process (Fig. 1- Sample B). XRD analysis shows several peaks at different 2θ angle values, which indicates the presence of polycrystalline structures of titania, including the main anatase phase at 2θ angle of 25^o^, as well as molybdenum oxides at 2θ angles of 48^o^ and 54^o^ (Fig. 2) [8], [9], [10].

**Fig. 3:**
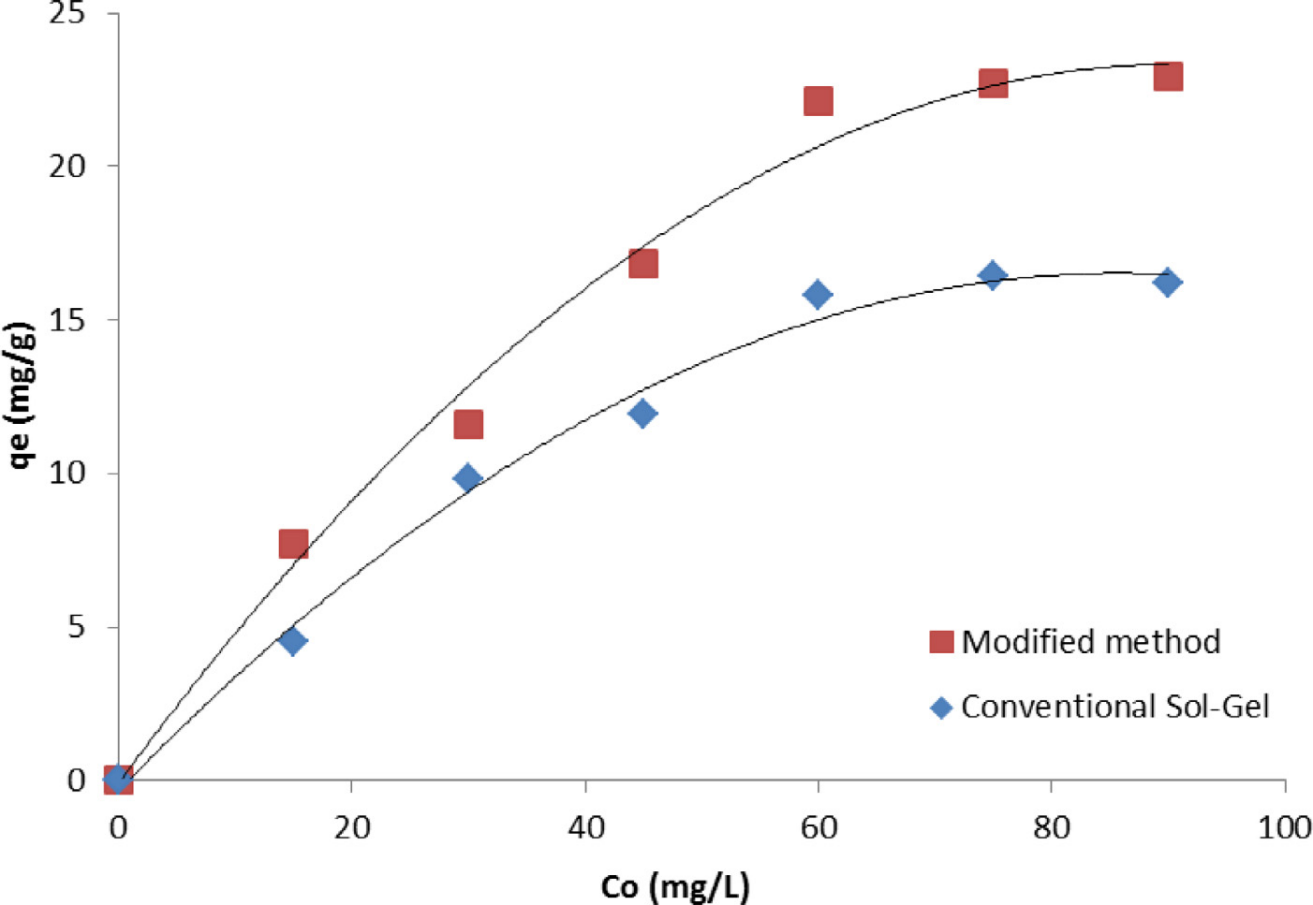
Photocatalytic degradation of MB using MoO_3_-TiO_2_ prepared via modified and conventional sol-gel methods.

The catalyst performance for the photocatalytic degradation of methylene blue (MB) inaqueous solutions was tested. During catalytic tests, six solutions of MB with initial concentrations (C_0_) ranging from 15 to 90 ppm were prepared, and each solution was divided into two portions. Subsequently, 0.5 g of each MoO_3_-doped TiO_2_ test sample (from the modified and conventional sol-gel method) was added to each portion. All samples were exposed to ultraviolet (UV) light for 18 h to ensure equilibrium was reached, and the final MB concentrations were measured using a UV-visible (Model: ShimadzuUV-Vis-3500) spectrometer. The catalyst uptake (q_e_) was calculated by comparing the initial and final MB concentrations, and the results showed an improved performance for the catalyst prepared by the modified sol-gel method, as shown in Fig. 3.

The improvements in morphology and the photocatalytic performance of the catalyst produced via the modified approach may be attributed to the enhanced temperature control of the reaction and the prevention of agglomeration using ultrasonication and alcohol separation in the modified sol-gel method [11], [12], [13], [14], [15].

Furthermore, the resulting product may be applied in many other physical and chemical processes, including the reduction of heavy metals in aqueous solutions, coating of optical and non-optical films, and as a catalyst support. The synthesis method may be tailored to achieve specific properties according to the type of application. The proposed method can be effectively altered to obtain desired products by varying parameters, such as the amount of precursor, acid, and water as well as the frequency and duration of ultrasonication and stirring speed, which play a role in the determining the properties of the resulting product [3,12].

## Declaration of Competing Interests

The authors declare that they have no known competing financial interests or personal relationships that could have appeared to influence the work reported in this paper.
